# Continuing Cetuximab *vs* Bevacizumab plus chemotherapy after first progression in wild-type *KRAS*, *NRAS* and *BRAF V600E* metastatic colorectal cancer: a randomized phase II trial

**DOI:** 10.7150/jca.60014

**Published:** 2021-06-26

**Authors:** Danyang Li, Feng Wang, Shuning Xu, Ke Li, Xiangrui Meng, Yangyang Huang, Ning Ma, Lei Qiao, Gaizhen Kuang, Jinghong Chen, Ying Liu

**Affiliations:** 1 Department of Oncology, Affiliated cancer hospital of Zhengzhou University, Henan Cancer Hospital, Zhengzhou, China 450008.; 2 Department of Oncology, The first affiliated hospital of Zhengzhou University, Zhengzhou, China 450052.; 3 Department of Oncology, Henan Province Hospital, Zhengzhou, China 450003.

**Keywords:** metastatic colorectal cancer, Cetuximab first progression, RAS wild type, MET expression

## Abstract

To evaluate the clinical efficacy of continuing cetuximab vs bevacizumab plus chemotherapy crossover after first progression to cetuximab regimen in wild-type *KRAS*, *NRAS* and *BRAF V600E* mCRC, we conducted this prospective, open-label and randomized phase 2 trial in three cancer centers from Oct 1, 2016 to July 1, 2020. Eligibility criteria included documented progressive disease during or after first-line treatment with cetuximab regimen; second biopsy confirmed as *KRAS*, *NRAS* and *BRAF V600E* wild-type mCRC. Patients were randomized to arm A (cetuximab+chemo) or arm B (bevacizumab+chemo) with second-line chemotherapy crossover. The primary end point was progression free survival (PFS). Secondary end points included objective response rate (ORR), overall survival (OS) and toxicity. Tissue VEGFA, ERBB2 and MET mRNA were examined by real time RT-PCR.

A total of 104 patients (53 in arm A and 51 in arm B) were enrolled. Median PFS was 7.7 months (95% CI: 6.5-8.9) for arm A and 6.3 months (95% CI: 4.5-8.1) for arm B (p=0.931). Median OS was 18.2 months (95% CI: 14.5-21.9) for arm A and 16.4 months (95% CI: 14.2-18.6) for arm B (p=0.339). The ORR was 28.3% and 19.6% in arm A and arm B (p=0.31), respectively. MET mRNA was highly expressed in the cetuximab-progressed tumors, but treatment responsiveness to cetuximab or bevacizumab in each arm was not correlated with the MET expression level. The results showed no significant difference in PFS, OS and ORR between the two arms, but a trend in favor of the cetuximab continuation plus chemotherapy crossover was examined in all end points. High expression of MET in cetuximab-progressed tumors may indicate an existence of MET-dependent tumor cell population.

## Introduction

Colorectal cancer (CRC) is one of the commonly diagnosed cancers and a leading cause of cancer-related death throughout the world 1. Chinese patients account for nearly one tenth of the global CRC burden 2. Both CRC incidence and death have significantly increased in the past two decades in China, in parallel with rapid economic growth, and continue to rise 2. In comparing with developed countries, Chinese CRC patients have a poorer prognosis as 50-75% patients are initially diagnosed with stage III-IV diseases 3. Indeed, CRC is emerging as a major healthcare challenge in China.

For patients with metastatic CRC (mCRC), treatment regimens including multiple chemotherapy and targeted agents have significantly evolved over the recent ten years 4-7. Currently there are 3 major therapeutic drug classes for mCRC treatment: cytotoxic chemotherapy combinations (e.g., fluorouracil and folinic acid combined with oxaliplatin (FOLFOX) or irinotecan (FOLFIRI)), anti-epidermal growth factor receptor (EGFR) antibodies (e.g., cetuximab), and anti-vascular endothelial growth factor (VEGF) inhibitors (e.g., bevacizumab). Addition of cetuximab or bevacizumab to cytotoxic chemotherapy combinations has been approved to provide more clinical benefits in first-line treatment of mCRC than chemotherapies alone 8, 9. Although there were inconsistent data on which is the optimal choice of the first-line targeted therapy 10, 11, recent studies in analyzing 2,576 mCRC patients from two randomized controlled trials and three prospective cohorts indicate that cetuximab provides better clinically relevant effects than bevacizumab 12. However, for patients fail to the first-line cetuximab plus chemotherapy treatment, benefits of further-line treatment still warrants clinical investigation. Especially, following disease progression, many patients have a good performance status and are willing to receive further treatment.

In addition to the sequential therapeutic schedule of offering maintenance therapy and reintroduction of chemotherapy regimens to patients with nonresectable mCRC 13-15, multiline treatment strategy of continuing bevacizumab after progression to first-line bevacizumab plus chemotherapy demonstrated optimal clinical benefits in prolonging progression-free survival (PFS) and overall survival (OS) in mCRC patients 7, 16, 17. In lieu of this, continuing cetuximab after progression to first-line cetuximab may also be promising. The underlying hypothesis is that a sustained inhibition of EGFR signaling with cetuximab would continuously eliminate sensitive clones of RAS wild-type tumor 18. In addition to RAS mutation, other resistant mechanism of mCRC to anti-EGFR antibodies includes the aberrant VEGF signaling 19, i.e., a higher tumor VEGF expression is associated with worse overall survival in mCRC patients treated with anti-EGFR antibodies 20. In considering that VEGF is continuously expressed throughout tumor progression in facilitating tumor angiogenesis 21, an interesting question thus emerges: for patients that fail to the cetuximab-based first-line combination treatment, which is an optimal choice of further-line treatment: cetuximab continuation? Or switch to bevacizumab?

This randomized phase 2 trial compared standard chemotherapy combined with either cetuximab or bevacizumab in mCRC patients with wild-type *KRAS*, *NRAS* and *BRAF V600E* tumors that had progressed after cetuximab first-line regimen.

## Methods

### Patients

One hundred and thirty-eight sporadic colorectal cancers patients were evaluated for enrollment in this trial from Oct 1, 2016 through July 1, 2020 in 3 hospitals in Henan Province of China. Inclusion criteria included ≥18 years old; confirmed as adenocarcinoma of the colon or rectum with measurable metastasis; the second biopsy confirmed as KRAS (exon 2, 3, 4), NRAS (exon 2, 3, 4) and BRAF V600E wild-type; Eastern Cooperative Oncology Group (ECOG) performance status 0-1; documented progressive disease (PD) during or after first-line treatment with cetuximab plus standard chemotherapy; normal organ and bone marrow function. This trial was approved by the ethics committee of Henan Cancer Hospital (IRB#2016ct084). Written informed consent were obtained from all patients.

### Interventions and Randomization

This trial was an open-label and 1:1 randomized phase 2 trial in assessing 2 standard regimens: cetuximab or bevacizumab, combined with FOLFOX or FOLFIRI chemotherapy after failed to first-line treatment containing cetuximab. FOLFOX or FOLFIRI crossover was adopted. Cetuximab 500mg/m^2^ per 2 weeks (arm A) or bevacizumab 2.5 mg/kg per week equivalent (arm B), was administered with FOLFOX or FOLFIRI until disease progression, occurrence of unacceptable toxic effects, or patient's refusal. Randomization was stratified by first-line chemotherapy, PFS with the first-line therapy (≤9 months vs >9 months), and the center.

### Study End Points and Assessments

The primary end point was progression free survival (PFS), defined as the time from randomization to disease progression or death from any cause whichever occurred earlier. Secondary objectives were median overall survival (OS), objective response rates (ORRs) measured by RECIST 1.1, and safety by Common Terminology Criteria for Adverse Events, version 4.03. Tumor response was evaluated at baseline and every 6 weeks until disease progression. Follow-ups were conducted every 3 months for treatment related serious adverse effects (AEs); subsequent anti-cancer therapy; and survival time.

### Tissue Samples and Molecular Analysis

Tumor biopsy tissues from all enrolled patients, of which 22 had a paired normal mucosa sample taken 5 cm from the primary tumor, were snap-frozen and stored in liquid nitrogen until DNA and RNA extraction. QIAamp DNA Mini Kit and RNeasy Mini Kit (Qiagen) were used for extracting DNA and RNA, respectively. Gene mutations were examined using KRAS/BRAF Mutation Analysis Panel Kit and NRAS Mutation Analysis Kit (KRAS exons 2, 3, 4 and BRAF V600E, NRAS exons 2, 3, and 4; EntroGen). These analyses, approved for *in vitro* diagnosis, use allele-specific PCR probes to identify 18 mutations of KRAS, 11 mutations of NRAS, and BRAF V600E mutations, with detection limit <1%. mRNA expressions of VEGFA, ERBB2 and MET were examined by real time RT-PCR using primers as below: sense CCATCCTGTGTGCCCCTGAT and anti-sense GCTGGCCTTGGTGAGGTTTG for VEGFA; sense TGGAACACAGCGGTGTGAGAA and anti-sense TTGCAGCCAGCAAACTCCTG for ERBB2; sense TGTGCATGAAGCAGGAAGGAACT and anti-sense AGCTGTTGCAGGGAAGGAGTG for MET. GAPDH served as an internal control for the real time RT-PCR analysis.

### Statistical Analysis

A modified intention-to-treat (mITT) analysis for the primary end point was performed, including all enrolled patients who received at least 1 dose of study drug. Sample size calculation for PFS was performed using a two-tail test in PASS 2020. The sample size obtained, using an alpha risk of 0.05, a beta risk of 0.30, the Control Group PFS of 4.5 months and the Experimental Group PFS of 7.5 months with none follow-up loss, was 51 subjects per group in each arm. PFS and OS (with their 95% CIs) were summarized using the Kaplan-Meier method. Log-rank test was used to evaluate treatment efficacy and to account for the 3 stratification factors. Hazard ratios (HRs) and the 95% CIs were determined using Cox proportional hazards regression models. ORR was analyzed using a Fisher exact test between the 2 arms; ORR estimates and 95% Wilson CIs were presented. Analyses were performed using SAS version 9.4 (SAS Institute Inc).

## Results

### Patient characteristics

**Figure 1:**
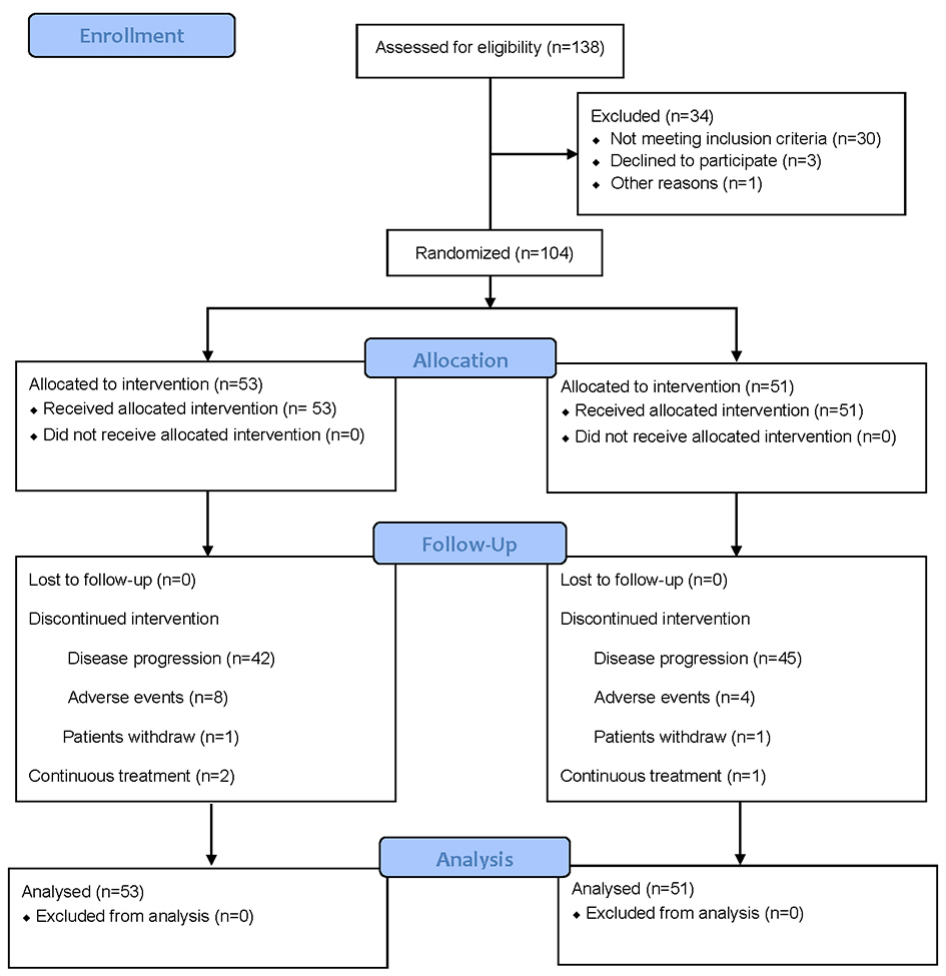
CONSORT 2010 Flow Diagram for patient enrollment and study design.

From Oct 1, 2016 through July 1, 2020, 104 out of 138 evaluated patients were enrolled at 3 cancer centers in Henan province of China as the mITT population (Figure 1). Patient characteristics and the use of chemotherapy were distributed similarly in each arm (Table 1). In the study, 49 patients who received the FOLFIRI regimen in first-line switched to the FOLFOX regimen and vice versa for 55 patients from FOLFOX regimen in first-line to the FOLFIRI regimen. Tumor biopsies were examined as wild-type *KRAS* (exon 2, 3, 4), *NRAS* (exon 2, 3, 4) and *BRAF V600E* for all patients included in this study.

### Efficacy

**Figure 2:**
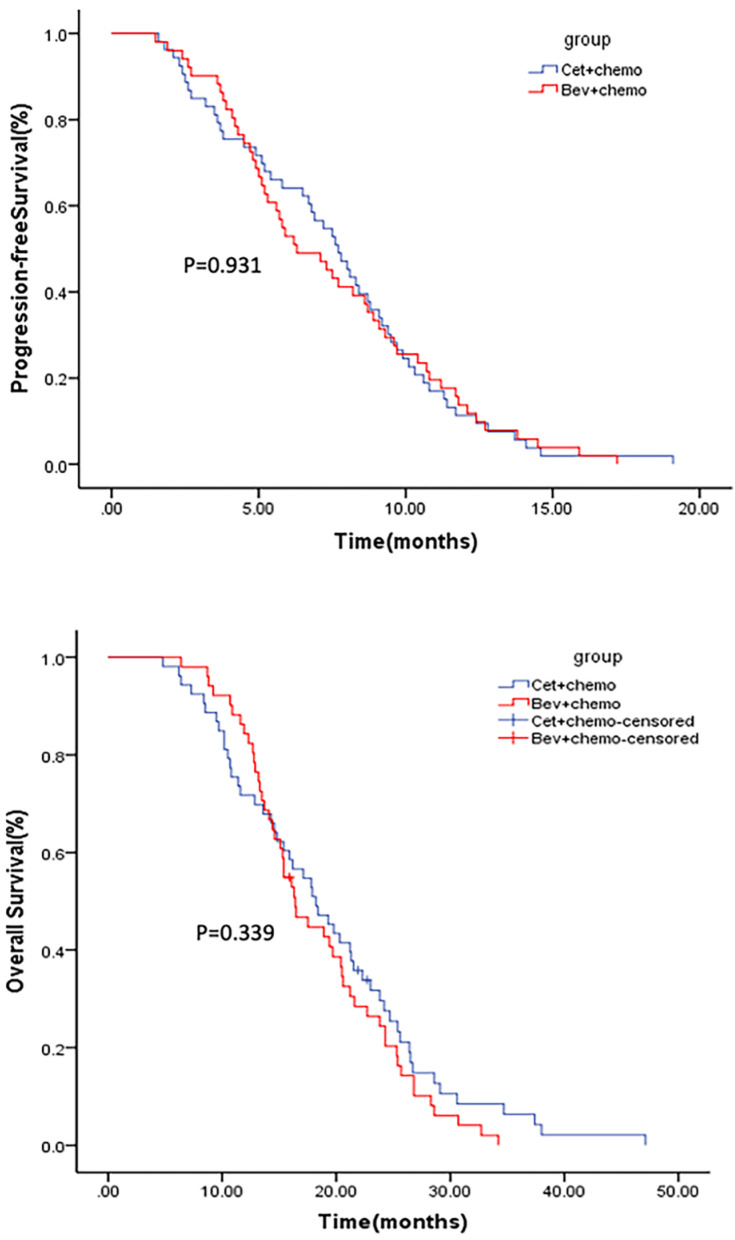
Kaplan-Meier estimates of PFS (upper) and OS (lower).

The median follow-up for the mITT population was 38 months (1-48 months). Median PFS was 7.7 months (95% CI 6.5-8.9) for arm A and 6.3 months (95% CI: 4.5-8.1) for arm B (p=0.931) (Figure 2). Median OS was 18.2 months (95% CI: 14.5-21.9) for arm A and 16.4 months (95% CI: 14.2-18.6) for arm B (p=0.339) (Figure 2). The ORR was 28.3% and 19.6% in arm A and arm B (p=0.31), respectively. Subgroup analysis conducted in patients with early tumor shrinkage (ETS) or achieved a PFS >9 months during first-line therapy, and those with left-sided primary tumors, PFS and OS were similar in patients treated in arm A and arm B (Supplementary Figure 1-3). Multivariable analysis showed that ECOG PS 0 (p=0.001), primary tumor resection (p=0.000), first-line PFS >9 months (p=0.008) and further anticancer therapy (p=0.001) contributed significantly to the improved overall survival of the mCRC patients (Table 2).

### Tolerability

Enrolled patients received a median number of 16 treatment cycles (3 to 37 cycles in arm A, and 4 to 38 cycles in arm B). At least 1 AE was reported for 48 out of 53 patients (90.6%) in arm A and 45 of 51 patients (88.2%) in arm B. Grade 3 and 4 AEs occurred in 17 of 53 patients (32.1%) in arm A and 15 of 51 patients (29.4%) in arm B (Table 2). The most frequently grade 3-4 AEs observed in arm A and arm B were leucopenia (17.0% vs 19.6%), diarrhea (9.4% vs 13.7%), and hand-foot syndrome (7.5% vs 5.9%). 15.1% patients experienced grade 3 rash in arm A, while 9.8% patients were observed with grade 3 hypertension in arm B. No toxic deaths were reported in either arm.

Chemotherapy was discontinued due to AEs in 4 patients (7.5%) in the cetuximab arm and 3 patients (5.9%) in the bevacizumab arm. Cetuximab discontinuation owing to AEs happened in 8 patients (15.1%), and bevacizumab discontinuation owing to AEs happened in 4 patients (7.8%).

### Molecular association to efficacy

**Figure 3:**
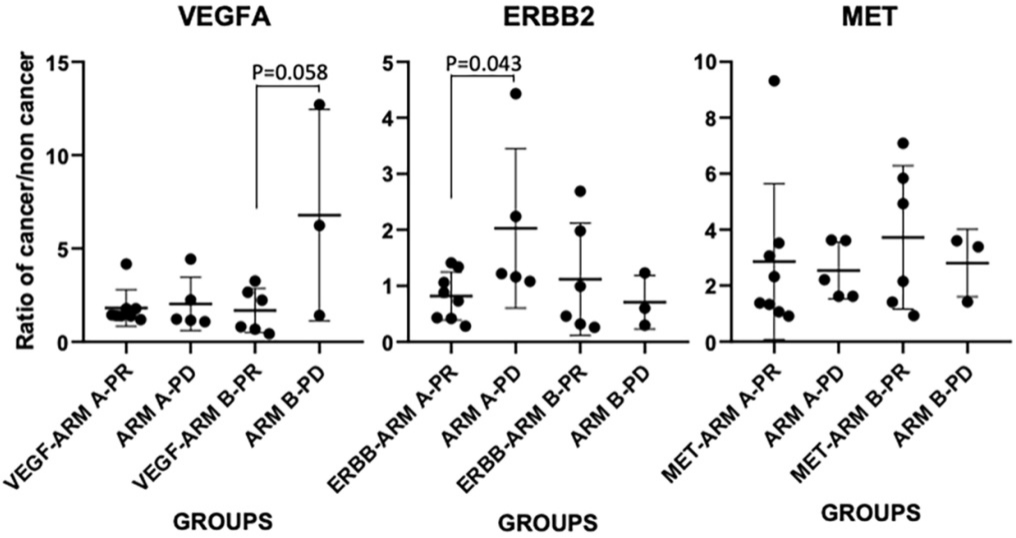
mRNA expressions of VEGFA, ERBB2 and MET in cancerous vs non-cancer normal tissue (ratio of cancer/non cancer) in each treatment arm and patients with PD or PR responsiveness.

Upon examining the mRNA expressions of VEGFA, ERBB2 and MET in cancerous vs non-cancer normal tissue (n=22), we first confirmed that between the two arms, expressions of these three markers in patients' tumor (normalized to paired normal tissue) didn't have any significant difference as the baseline, i.e., the p values for VEGFA, ERBB2 and MET mRNA expressions between the two arms are 0.81, 0.46 and 0.56, respectively (n=13 in arm A, n=9 in arm B). However, tumor VEGFA mRNA and MET did have a 2.5±2.67-fold and 3.02±2.16-fold increase in comparing to that in the normal tissue in the patients, while the expression of ERBB2 mRNA showed a slightly decrease in the tumor vs non-tumor tissue (0.93±0.69-fold change). We further compared their expressions in each arm between treatment responsive vs non-responsive patients (Figure 3). In arm A, 8 patients showed partial response (PR) and 5 had PD, in arm B 6 showed PR and 3 had PD. In arm B patients, VEGFA showed an elevated expression in PD tumors (6.78±5.67) vs PR tumors (1.68±1.19) (p=0.058). In arm A patients, ERBB2 showed an elevated expression in PD tumors (2.03 ±1.55) vs PR tumors (0.82±0.43) (p=0.043). There was no significant difference for MET expression in each arm between PR and PD tumors.

## Discussion

This is a novel trial in evaluating the clinical benefits of continuing EGFR inhibition versus VEGF inhibition in treating wild-type *KRAS*, *NRAS* and *BRAF V600E* mCRC tumors with second-line cetuximab or bevacizumab plus chemotherapy crossover after tumor progression to first-line cetuximab regimen. From the results on the 104 ITT cohort, no statistical difference in PFS, OS and ORR were observed among the two arms. However, the better numerical values of all the end points seem favor the cetuximab continuation plus chemotherapy crossover.

Previous trials have shown clinically therapeutic benefits in continuing cetuximab (the CAPRI-GOIM trial) 22 or continuing anti-angiogenic drug (the ML18147 and RAISE trials) 16, 23 after first progression than chemotherapy alone in mCRC. The recent PRODIGE18 trial 7 suggested a favorable efficacy of continuation with bevacizumab vesus switching to cetuximab plus chemotherapy after first progression in mCRC patients. Together with the current study that cetuximab continuation plus chemotherapy after first progression had favorable PFS, OS and ORR than switching to bevacizumab, these results indicate that the choice of first-line targeted therapy is relevant for further course of disease 24. In the FIRE-3/AIO KRK0306 trial 25 in which subsequent-line therapy was evaluated that was not part of the previous regimen in KRAS wild-type mCRC, first-line application of anti-EGFR targeted therapy seems optimal for effective subsequent therapy including anti-angiogenic agents.

In our study, although we examined an increased expression of VEGFA in the cetuximab-progressed *KRAS*, *NRAS* and *BRAF V600E* wild-type mCRC tumors, our study didn't find clinical advantage of the anti-VEGF agent bevacizumab in conferring improved PFS and OS versus cetuximab continuation. This provides first and important clinical evidence that cetuximab treatment in first and second line in combination with crossover chemotherapy could be an effective option in comparable to cetuximab in first and bevacizumab as second line treatment, for patients with wild-type *KRAS/NRAS/BRAF* tumors. Overall, our data also suggest that mutation classification for the EGFR downstream *KRAS/NRAS/BRAF* genes would identify EGFR-dependent tumors that are highly possible in responding to anti-EGFR treatment beyond progression.

Potential mechanisms of CRC cells become resistant to anti-EGFR therapy have been extensively investigated, particularly the activation of growth factor receptors MET and ERBB2 26. In the present study, 22 paired tumor and non-tumor normal tissue were examined for mRNA expressions of MET and ERBB2. MET mRNA showed a 3-fold increase in the tumor vs non-tumor normal tissue (3.02±2.16), while the expression of ERBB2 seems no change (0.93±0.69). Although MET was highly expressed in the cetuximab-progressed tumors, treatment responsiveness to cetuximab or bevacizumab in each arm was not correlated with the MET expression level, indicating that a MET-dependent tumor cell population may exist. Bardelli et al 27 reported the presence of rare *MET*-amplified tumor cells in some CRC patients before treatment with cetuximab, and cetuximab therapy acted as a selective pressure to expand this minor tumor cell population. Furthermore, interception of MET signaling could largely impair tumor growth in *MET*-amplified CRC 27. Together with these findings, our study may indicate that MET inhibitors in combination with cetuximab continuation could serve as a novel therapeutic opportunity to the patient population in our current study setting.

We are aware of the small sample size of the clinical study, especially the limited number of paired tissue specimens for the VEGFA, ERBB2 and MET mRNA examination in correlating to treatment response, thus larger cohort study is warranted. In addition, animal study in exploring the combination of EGFR inhibitor with MET inhibitor on EGFR treatment progressed CRC tumors may provide translational relevance.

In conclusion, in this randomized phase II study, cetuximab continuation vs bevacizumab plus chemotherapy crossover did not have significant difference in PFS, OS and ORR in *KRAS/NRAS/BRAF* wild-type mCRC patients who have progressed to cetuximab regimen. However, a trend in favor of the cetuximab continuation was examined in all end points. MET was highly expressed in the cetuximab-progressed tumors, but treatment responsiveness to cetuximab or bevacizumab in each arm was not correlated with the MET expression level, indicating that a MET-dependent tumor cell population may exist; MET inhibitors in combination with cetuximab continuation could serve as a therapeutic opportunity to these patients.

## Supplementary Material

Supplementary figures.

## Figures and Tables

**Table 1:** Demographic and clinical characteristics of patients at baseline

	Cet+chemo (N=53)	Bev+chemo (N=51)	*P*
**Sex**			
Male	31 (58.5%)	31 (60.8%)	0.844
Female	22 (41.5%)	20 (39.2%)	
Age (years)	56 (18-75)	59 (24-74)	
**ECOG performance status**			
0	38 (71.7%)	33 (64.7%)	0.529
1	15 (28.3%)	18 (35.3%)	
**Primary tumor location**			
Left-side colon	38 (71.7%)	39 (76.5%)	0.657
Right-side colon	15 (28.3%)	12 (23.5%)	
Primary tumor resection	38 (71.7%)	33 (64.7%)	0.527
**Histologic differentiation**			
Grade 1 or 2	8 (15.1%)	12 (23.5%)	0.325
Grade 3 or 4	45 (84.9%)	39 (76.5%)	
**Site of tumor metastasis**			
Liver	29 (54.7%)	30 (58.8%)	0.697
Lung	23 (43.3%)	26 (50.9%)	0.556
Lymph node	32 (60.4%)	24 (47%)	0.238
Bone	13 (24.5%)	9 (17.6%)	0.474
Peritoneal	8 (15%)	11 (21.5%)	0.453
Others	6 (11.3%)	5 (9.8%)	0.386
**First-line chemotherapy**			
Irinotecan-based	26 (49.1%)	23 (45.1%)	0.699
Oxaliplatin-based	27 (50.9%)	28 (54.9%)	
First-line early tumor shrinkage	18 (34.0%)	14 (27.5%)	0.528
**First-line progression-free survival (months)**			
≤9	22 (41.5%)	19 (37.3%)	0.692
>9	31 (58.5%)	32 (62.7%)	
Cetuximab maintenance therapy after first-line chemotherapy	33 (62.3%)	32 (62.7%)	1.000

**Table 2:** Multivariable analysis of factors contributed to the patients' overall survival

Variable factors	Multivariable analysis (N=104)	
	OS (months)	*P*
Gender (Male vs. Female)	17.9 vs. 16.4	0.48
Primary tumor location (left vs. right)	17.5 vs. 17.1	0.911
ECOG PS (0 vs. 1)	20.6 vs. 12.8	0.001
Primary tumor resection (Yes vs. NO)	21.2 vs. 12.9	0.000
First-line PFS (≤9 months or >9 months)	14.8 vs. 20.3	0.008
First-line early tumor shrinkage (Yes or NO)	21.2 vs. 15.1	0.002
Further anticancer therapy (Yes or NO)	22.3 vs. 12.8	0.001
