# Supplementary material for: Continuing Cetuximab vs Bevacizumab plus chemotherapy after first progression in wild-type KRAS, NRAS and BRAF V600E metastatic colorectal cancer: a randomized phase II trial
Source: J Cancer. 2021 Jun 26;12(17):5268–74. doi: 10.7150/jca.60014 (PMC8317523; doi:10.7150/jca.60014)
Supplement: Supplementary file 1 — Supplementary figures. [file jcav12p5268s1.pdf]

Continuing Cetuximab *vs* Bevacizumab plus chemotherapy after first progression in wild-type *KRAS, NRAS* and *BRAF V600E* metastatic colorectal cancer: a randomized phase II trial

Danyang Li[a], Feng Wang[b], Shuning Xu[a], Ke Li[a], Xiangrui Meng[b], Yangyang Huang[a], Ning Ma[c], Lei Qiao[a], Gaizhen Kuang[a], Jinghong Chen[a], Ying Liu[a,*]

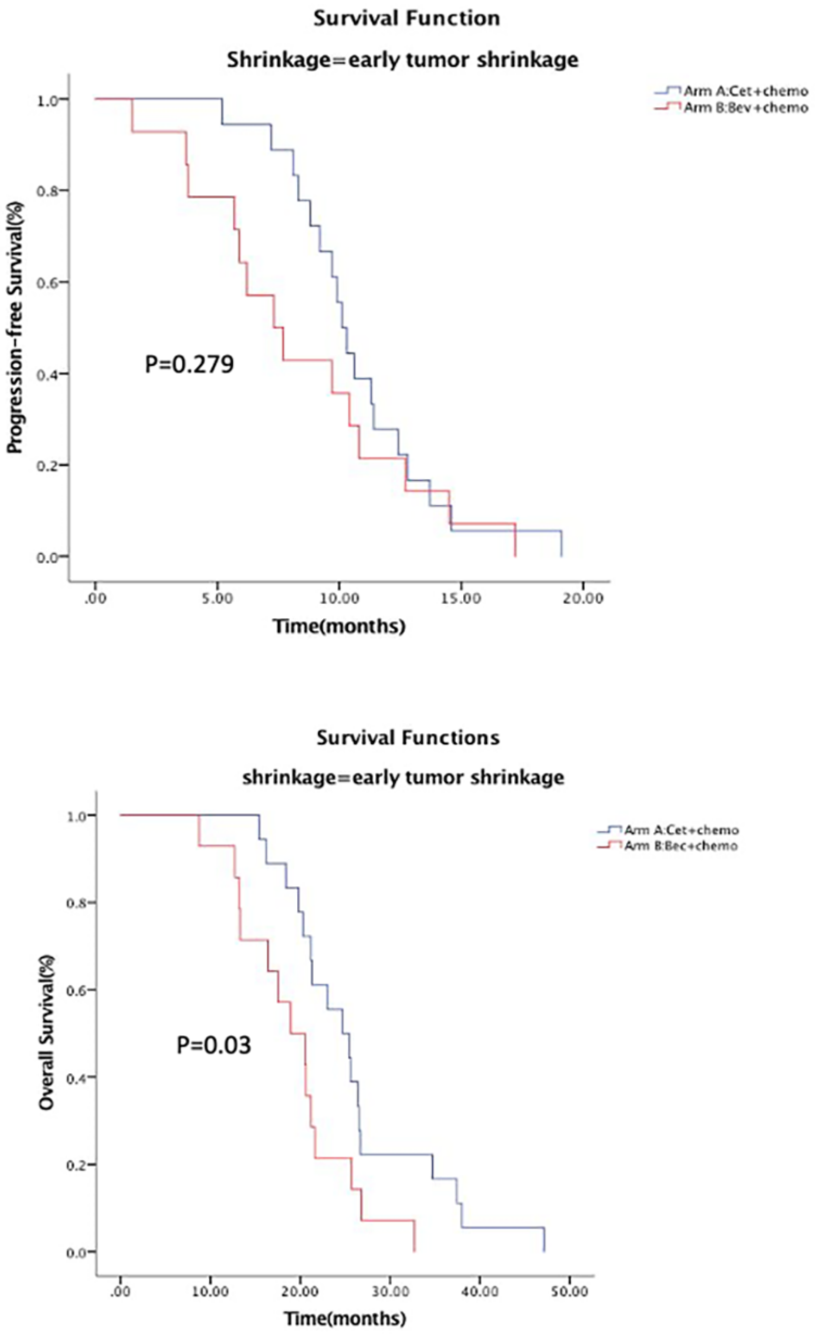

Sup Fig 1. Kaplan-Meier estimates of PFS (upper) and OS (lower) in patients with early tumor shrinkage.

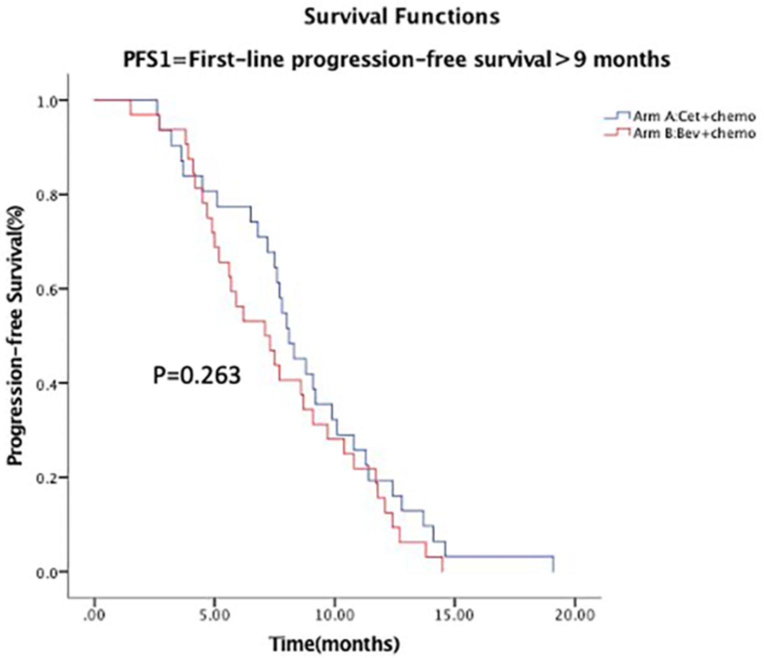

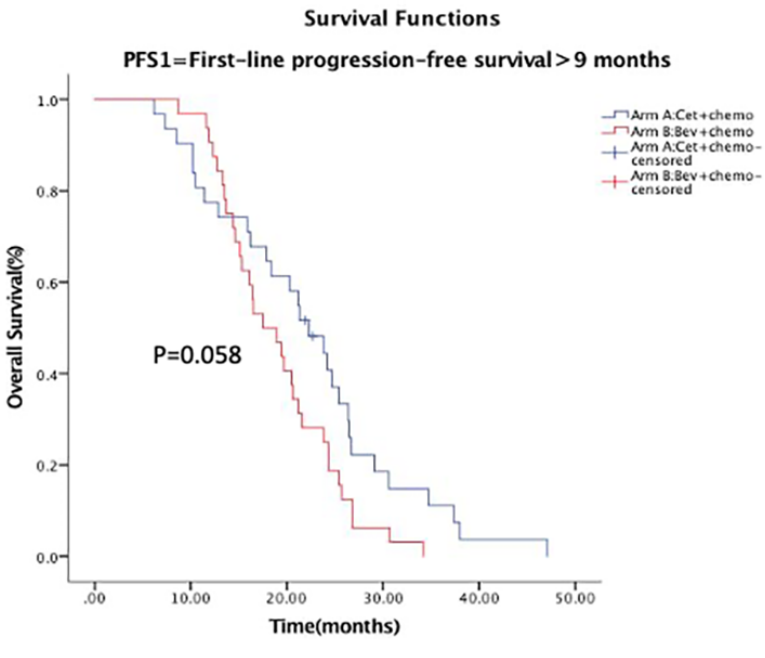

Sup Fig 2. Kaplan-Meier estimates of PFS (upper) and OS (lower) in patients with first-line PFS >9 months.

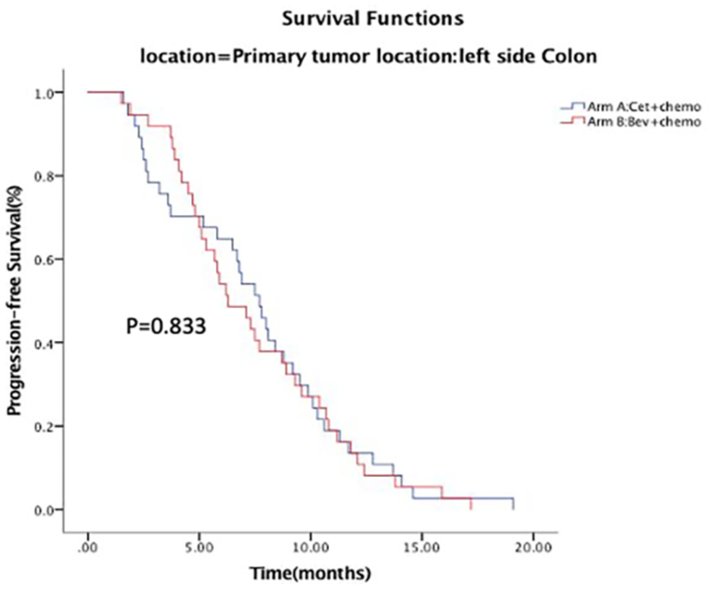

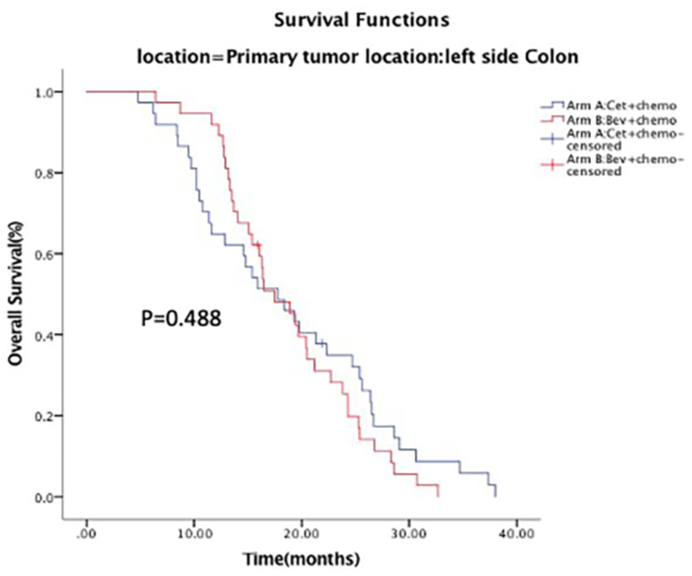

Sup Fig 3. Kaplan-Meier estimates of PFS (upper) and OS (lower) in patients with left-sided primary tumors.
